# Low cost, high performance ultrafiltration membranes from glass fiber-PTFE–graphene composites

**DOI:** 10.1038/s41598-020-78091-x

**Published:** 2020-12-03

**Authors:** Umar Khan, Sonia Biccai, Conor S. Boland, Jonathan N. Coleman

**Affiliations:** 1 School of Science, Institute of Technology, Sligo, Ash Lane, Sligo, Ireland; 2 School of Physics, CRANN and AMBER Research Centres, Trinity College Dublin, Dublin 2, Ireland

**Keywords:** Materials science, Nanoscience and technology

## Abstract

The development of low-cost ultrafiltration membranes with relatively high flow rate and selectivity is an important goal which could improve access to clean water in the developing world. Here we demonstrate a method to infuse mixtures of graphene nanosheets and Teflon nanoparticles into ultra-cheap glass fibre membranes. Annealing the resultant composites leads to coalescence of the Teflon, resulting in very stable membranes with significantly enhanced mechanical properties. In filtration tests, while adding ~ 10 wt% graphene/Teflon to the glass fibre membrane decreased the flow rate by × 100, the selectivity improved by × 10^3^ compared to the neat glass fibre membrane. This combination of selectively and flow rate was significantly better than any commercial membrane tested under similar circumstances. We found these membranes could remove > 99.99% of 25–250 nm diameter SiC nanoparticles dispersed in ethanol, transmitting only particles with diameters < 40 nm, performance which is superior to commercial alumina membranes. Field trials on dirty canal water showed these composite membranes to remove aluminium to a level × 10 below the EU limit for drinking water and reduce iron and bacteria contents to below detectable levels.

## Introduction

Access to clean water is one of the greatest problems currently facing humanity. Globally, approximately two billion people use contaminated drinking water daily^1^ while another 750 million people do not have reliable access to drinking water^2^. As a result, over three million people die annually due to water-related illnesses^3^. While access to clean drinking water is not usually a problem in the developed world, demands for water are growing rapidly. It is estimated that industry is responsible for ~ 40% of demand for water in the EU^4^. The global demand of industrial water is expected to increase by 400% by 2050^2^. Clearly, solutions must be found to meet the demand for both drinking and industrial water. One important solution to both these problems is to use filtration to purify both dirty and waste water^5^.

Water purification by filtration typically uses porous, permeable membranes to separate dispersed solids such as particles, macromolecules and micro-organisms from the water. In general, the water to be treated is passed through the membrane resulting in solids above a certain cut-off size remaining in the retentate while the purified water (containing only solids smaller than the cut-off size) passing through the membrane. The cut-off size depends on the pore size distribution within the membrane. In all cases, membranes need to maximise both selectivity (i.e. the ability to remove as much impurity material as possible) and flow rate (the rate at which purified water can be produced). In addition, membranes material must be mechanically robust, chemically stable and cost effective.

A number of different membranes types are widely used. Fibrous membranes, are generally polymeric in nature and are commonly made using electrospinning^6–8^. These membranes often suffer from bio and chemical fouling both on the surface and deep inside the structure^9,10^, and are limited by the mechanical, thermal and chemical instabilities typical of polymers^11^. Similar to fibrous membranes are foam structures which are also polymeric and often fabricated from cellulose acetate or nylon. As a result, these membranes tend to suffer from the same problem/solutions as fibrous membranes. Ceramic membranes tend to be fabricated from alumina or other metal oxides and are made using sol–gel techniques^12^. They have very good thermal stability but are brittle, have low acid/ base stability^13^ and can be very expensive.

More recently, attention has moved to nano-material-based membranes, often incorporating 1D^14,15^, or more relevant here, 2D materials such as graphene and graphene oxide (GO)^16–22^ or even 2D polymers^23^, for use in filtration. For example, a large number of papers have described GO membranes with very high selectivity for water purification^24–26^. In addition, a number of researchers have mixed graphene with polymer matrices, yielding highly selective filter membranes with enhanced mechanical properties^27,28^. Alternatively graphene oxide has been combined with inorganic nanoparticles, for example TiO_2_, in order to control the pore size^16^. Research in 2D materials for filtration has largely focused in high-performance applications such as desalination. However, although these membranes often show significant potential in such high-performance applications, more work is definitely needed on nano-enabled filters for relatively low-end applications such as water purification in the third world. In such fields, extremely high selectivity is perhaps less important than achieving scalability and low cost.

Thus, it is clear that there is an opening for a new low-cost, chemically and mechanically stable membrane material which can combine good flow rate and selectivity. When thinking of high-performance membranes, one does not usually think of glass fibre membranes. These are very low-cost membranes that consist of a non-woven network of glass fibres. While they display very high flow rates, very good thermal and chemical stability, they have poor mechanical properties and very low selectivity. Typically, they are generally used only as pre-filter^29,30^. However, we hypothesised that these low-cost membranes might be combined with a graphene-based nano-composite to develop membranes with reasonably high performance but low cost. Here we demonstrate a method to modify commercially available glass fibre membrane with solution processable composites of graphene and Teflon to prepare ultra-filtration membranes with tuneable properties. We find combinations of flow rates and selectivity superior to a range of commercial membranes.

## Results and discussion

### Composite membrane production and basic characterisation

**Figure 1:**
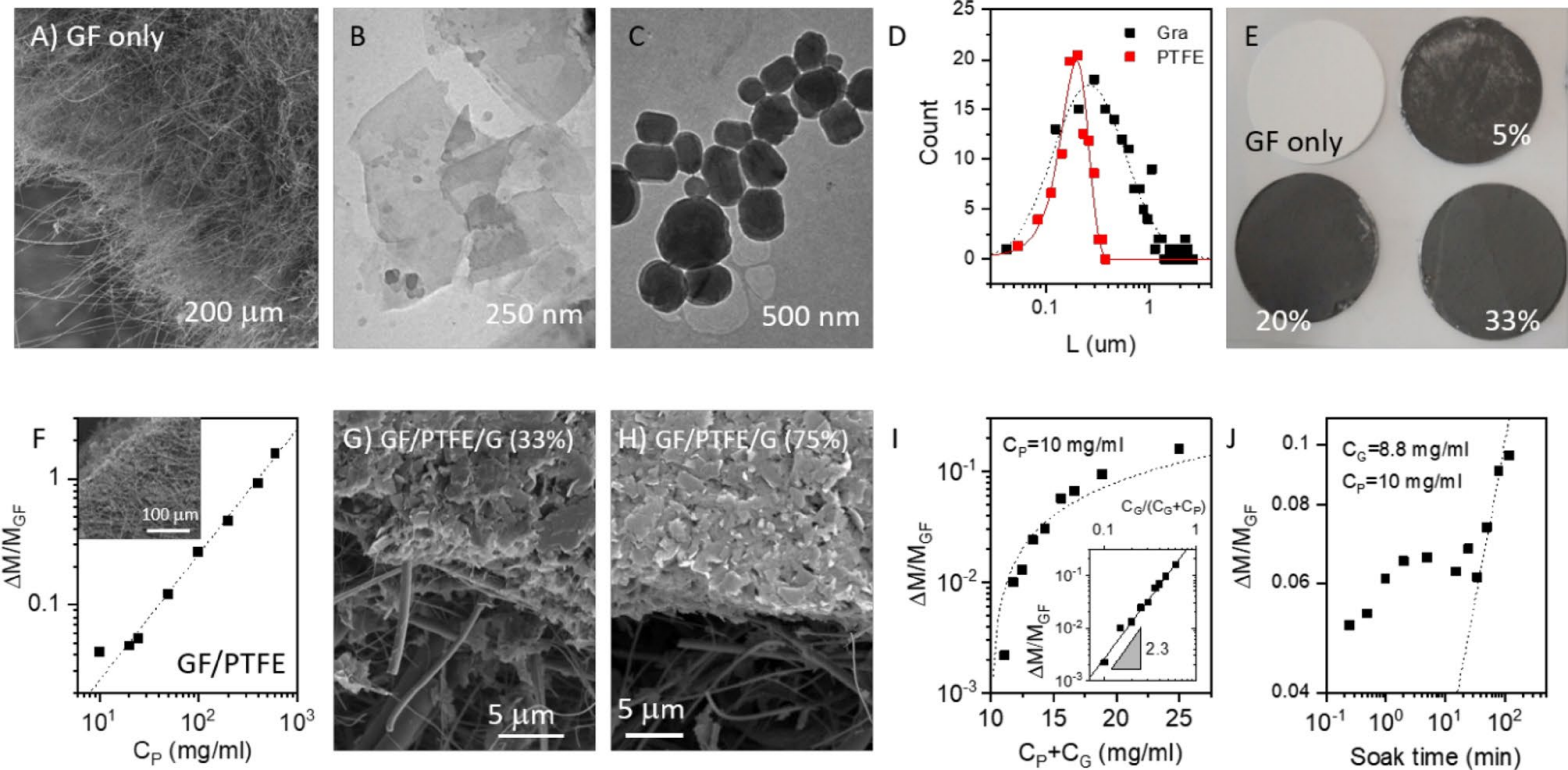
(**A**) SEM image of the edge of a glass fibre (GF) membrane. (**B**,**C**) TEM images of typical LPE graphene nanosheets (**B**) and PTFE nanoparticles (**C**). (**D**) Lateral size distribution for both graphene nanosheets and PTFE nanoparticles. (**E**) Photographs of GF membrane (top left) and membranes with a range of nominal PTFE/graphene contents. The number reflects the graphene content in the soak solution expressed as C_G_/(C_G_ + C_P_) where C_G_ and C_P_ are the concentrations of graphene and PTFE in the soak solutions. (**F**) The membranes are soaked in a dispersion of PTFE nanoparticles with the mass uptake plotted versus the concentration of PTFE (soak time 45 min). The dashed line represents linearity. Inset: SEM images of a GF membrane partially filled with PTFE. (**G**,**H**) GF membrane partially filled with PTFE and graphene where C_G_/(C_G_ + C_P_) = 33% (**G**) and 74% (**H**). (**I**,**J**) Graphs showing fractional mass uptake on soaking for two soaking scenarios: (**I**) The membranes are soaked in a dispersion of both PTFE nanoparticles and graphene nanosheets with C_P_ = 10 mg/ml and varying C_G_. The mass uptake is plotted versus the combined concentration of PTFE and graphene (soak time 45 min). The dashed line represents a linear increase in mass uptake with C_G_. Inset: Mass uptake poltted as a function of C_G_/(C_G_ + C_P_) showing a clear power law dependence. (**J**) The membranes are soaked in a dispersion of both PTFE nanoparticles and graphene nanosheets with C_P_ = 10 mg/ml, C_G_ = 8.8 mg/ml for varying soak times. The dashed line represents what would be expected if the uptake was solely diffusion limited.

Commercial glass fibre (GF) membranes are formed from mats of glass fibres, each fibre having diameter of a few microns and a length of many hundreds of microns. They have porosities of > 95% (i.e. densities of ~ 200 kg/m^3^), typical pore sizes of tens of micron and are very cheap to make (see Fig. 1A). However, because of the large pore sizes, although such filters do show high flow rates, they only block large particles. Here we aim to reduce the particle size transmitted through the membrane to the filtrate by using exfoliated graphene nanosheets to reduce the effective pore size. We reasoned that soaking the GF membranes in a dispersion of graphene nanosheets would lead to the infusion of nanosheets into the interior of the membrane resulting in the formation of a nanosheet network with small pore size. Importantly, such a network would be mechanically supported by the glass fibres.

To achieve this, we prepared few-layer graphene nanosheets (Fig. 1B) by liquid phase exfoliation^31,32^, a well-known graphene production technique (see “Methods”). While initial trials showed that graphene could be inserted by soaking the GF membrane in an aqueous, surfactant stabilised dispersion of graphene nanosheets, further experiments showed the graphene network to be very unstable, rapidly flaking of the membrane, especially during filtration trials. Early attempts to introduce a polymer binder by adding a soluble polymer to the graphene dispersion failed to give stable, robust membranes.

To address this problem, we used a novel solution to insert a polymeric binder into the GF membrane, either with or without the presence of graphene. Instead of using a soluble polymer as a binder, we used commercially available Teflon (PTFE) nanoparticles (PTFE-NPs). These nanoparticles are pseudo-spherical (Fig. 1C) and are supplied as an aqueous dispersion. TEM measurements showed both PTFE particles and graphene nanosheets to display a broad distribution of sizes typically in the range of hundreds of nm (Fig. 1D). The mean graphene nanosheet length was 625 nm while the mean PTFE nanoparticle diameter was 200 nm. To insert the graphene nanosheets and polymer binder into the GF membrane, we placed membranes into aqueous dispersions of either PTFE-NPs or mixtures of PTFE-NPs and graphene nanosheets and allowed them to soak in the liquid for various times. Under these circumstances, the liquid wicks into the porous interior of the GF membranes, bringing the dispersed material (i.e. PTFE-NPs/nanosheets) with it. Such wicking behaviour is essentially an example of capillary action^33^ and is driven by the favourable intermolecular interactions between the liquid and glass fibres. Over time both PTFE-NPs and graphene nanosheets become deposited onto the glass fibres, both at the surface and within the interior of the membrane, resulting in a significant mass uptake. Similar methods have been used to infuse polymer into carbon nanotube-based papers^34–36^. Such deposition was visible to the eye, with any membrane surface which was in contact with the graphene-containing dispersion changing from pure white to black (Fig. 1E). Sintering the resultant composite membranes at 420 °C resulted in very stable composites due to coalescence of the PTFE-NPs.

It was possible to control the mass uptake (ΔM) via either the concentration of the soaking dispersion or the soak time. Shown in Fig. 1F is data for the fractional mass uptake (ΔM/M_GF_ where M_GF_ is the initial mass of the GF membrane) of a GF membrane soaked in PTFE-NP-only dispersions of different concentrations, C_P_ (soak time 45 min). This data shows a clear linear increase of mass uptake with PTFE concentration. SEM imaging (inset) appeared to show PTFE inside the GF membrane after sintering, with the membranes displaying densities of ~ 210–850 kg/m^3^ consistent with significant porosity retention.

More importantly, we investigated the effect of soaking GF membranes in mixtures of graphene and PTFE-NPs with individual concentrations of C_G_ and C_P_ and so a combined concentration of C_G_ + C_P_. SEM images of composite membranes formed by soaking GF membranes in mixtures of graphene and PTFE are shown in Fig. 1G,H. These images show that, although some graphene/PTFE has entered the membrane, the majority has formed a thick layer on top. This shows that under these preparation conditions, the spatial distribution of infused material is non-uniform, with the local concentration of graphene/PTFE-NPs increasing from centre to surface. This observation is almost certainly due to deposition of graphene/PTFE-NPs onto the glass fibres which occurs preferential near the surface of the membrane resulting in the observed gradient of deposited graphene/PTFE-NPs.

We found it impossible to measure the individual masses of graphene and PTFE which had been taken up by the membrane. For example, thermogravimetric analysis failed because we were unable to completely burn off the graphene, possibly because of the poor thermal conductivity of the glass or the strong adhesion of the graphene. In addition, spectroscopic surface techniques such as XPS and Raman proved unsuitable as we cannot be sure that the graphene/Teflon ratio is the same at the membrane surface as in the bulk. This made it impossible to determine the mass of the individual components. As a result, we concentrate on the total mass uptake which could be found by weighing.

In Fig. 1I we plot the factional mass uptake (after sintering) as a function of combined PTFE and graphene concentrations. Here the PTFE concentration was fixed at C_P_ = 10 mg/ml while the graphene concentration, C_G_, was varied. Interestingly, for reasons we do not understand, the total mass uptake scaled roughly linearly with C_G_ (dashed line) suggesting the mass uptake to be dominated by graphene. However, it is clear that some PTFE does enter the membrane and act as a binder as such composite membranes are stable after sintering whereas those membranes prepared without PTFE (graphene only) were not. In the inset of Fig. 1I, we plot the mass uptake versus the mass fraction of graphene in the soak dispersion C_G_/(C_G_ + C_P_), finding a well-defined power law with exponent 2.3. This implies that graphene uptake is favoured over that of polymer and suggests the higher ΔM/M_GF_ dispersion may have a higher relative graphene constant than those membranes with lower mass uptake.

The fractional mass uptake is shown as a function of soak time in Fig. 1J for a fixed combination of graphene and PTFE soaking concentrations. Interestingly, over the first 10 min of soaking the mass uptake increases slowly before accelerating and increasing thereafter as $\Delta M/M_{GF} \propto \sqrt t$, in line with Washburn’s equation^33^, and consistent with diffusion limitations.

### Mechanical properties of membranes

**Figure 2:**
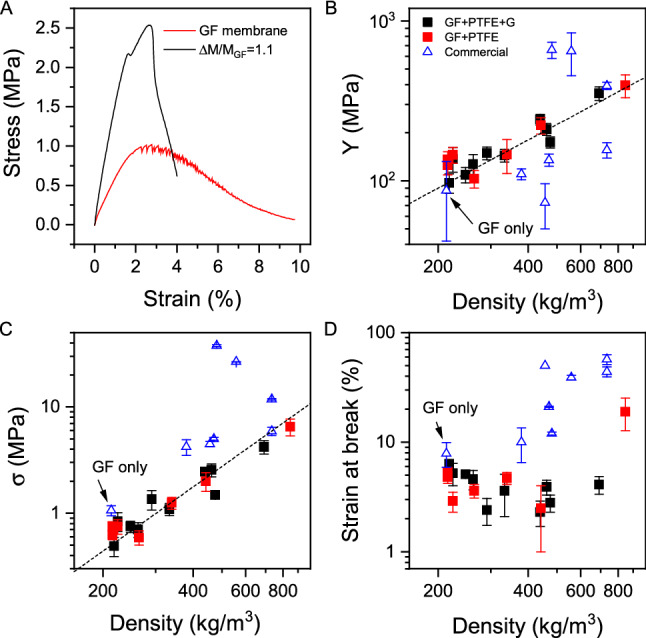
Mechanical properties of membranes. (**A**) Typical stress–strain curves for a GF membrane and a composite membrane with fractional mass uptake of 1.1. (**B**–**D**) Modulus (**B**), tensile strength (**C**) and strain at break (**D**), all plotted versus measure membrane density. Data is plotted for membranes filled with both PTFE and PTFE + graphene. Also shown are data for commercial membranes. The dashed lines in (**B**,**C**) represent linear and quadratic behaviour respectively.

For water purification applications, it is important that membranes are mechanically robust and not prone to cracking or other damage. It is possible that the addition of PTFE and graphene can actually improve the mechanical properties of the GF membranes. In particular, graphene is well known to improve the mechanical properties of composites under certain circumstances^37^. To test this, we performed tensile mechanical tests on membranes with a range of different values of ΔM/M_GF_. In addition, for comparison purposes, we performed the same tests on a number of commercial membranes including a GF-only membrane. Shown in Fig. 2A are representative stress–strain curves for a GF membrane (untreated) and a GF membrane soaked in graphene and PTFE-NPs with ΔM/M_GF_ = 1.1. It is clear from these curves that addition of PTFE and graphene improves the mechanical properties of the membranes.

To show this in more detail we extracted three parameters from the stress–strain curves, the modulus, Y, the tensile strength, σ, and the strain at break. We also measured the same parameters for membranes produced by infiltrating PTFE-NPs only into the GF membranes as well as a range of commercial membranes fabricated from teflon, polypropylene, polyester, nylon and cellulose. In order to facilitate comparison, we plot each mechanical property versus the measured density of the membrane under test, keeping in mind that for the composite membranes produced in this study, the density increases linearly with the mass uptake. Shown in Fig. 2B,C are the modulus, strength and strain at break plotted versus membrane density for all tested membranes. Both modulus and strength increase with membrane density and so with ΔM/M_GF_. In both cases, the data for GF membranes infiltrated with both PTFE-NPs alone and PTFE-NPs -graphene sit on the same trend line indicating the graphene to have little impact on the membrane mechanical properties (probably because most of the graphene is concentrated near the surface of GF membranes). Interestingly, both stiffness and strength follow different power laws with density consistent with $Y \propto \rho$ and $\sigma \propto \rho^{2}$ (dashed lines). While the strength-density scaling is in line with a number of papers on graphene-based aerogel or porous monoliths^38,39^, the modulus-density scaling is inconsistent with most published results which tend to show $Y \propto \rho^{2}$^39–41^. However, the structure of these composite membranes is quite complex with a spatially non-uniform distribution of infused particles so it is probably not surprising that the mechanics are atypical. While modulus and strength improve with infusion, the strain at break decreases slightly as shown in Fig. 2D, falling from ~ 8% in GF-only membranes to ~ 3% in highly-filled membranes. While this indicates an increase in brittleness, this is somewhat compensated by the increase in strength. We note that most of the commercial membranes showed combinations of modulus, strength and stiffness which were somewhat superior to our composite membranes. However, we note that these composite membranes are robust enough for practical use in filtration applications (see below) and are generally superior to GF-only membranes.

### Characterising GF/graphene/PTFE-NP composites as ultrafiltration membranes

We have tested composites of GF membranes infiltrated with both PTFE-NPs only as well as mixtures of PTFE-NPs and graphene as ultrafiltration membranes. We first performed experiments to determine whether any graphene leached out of the membranes during filtration. Extensive spectroscopic and microscopic analysis of filtrate showed no evidence of graphene, showing the nanosheets to be well bound to the membranes, probably due to the presence of the PTFE-NPs.

Ultrafiltration testing was performed via filtration experiments using dispersions of SiC nanoparticles in ethanol (0.2 mg/ml) as a feed dispersion. We chose SiC nanoparticles over more well-defined nano-particles because their broad size distribution (~ 50–200 nm in diameter) allows us to estimate the size distribution of particles transmitted through the membrane to the filtrate (see below). We filtered the SiC/ethanol dispersions through our composite membranes, measuring both the rate of permeate flow, F, (volume per second per unit area per unit pressure drop) through the membrane and the concentration of SiC in the filtrate (via optical absorbance spectroscopy). Dividing the latter quantity by the concentration of SiC in the initial dispersion (0.2 mg/ml) gave the mass fraction of SiC transmitted through the membrane (T) which is a measure of selectivity.

**Figure 3:**
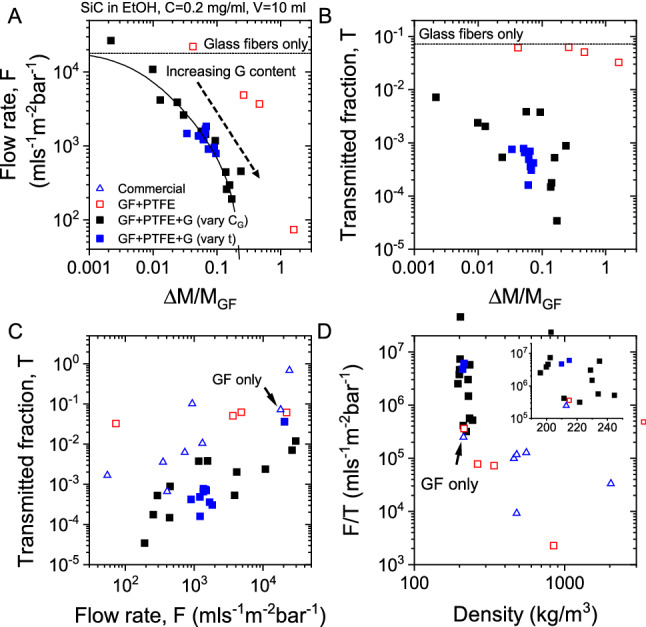
Filtration properties of nanocomposite membranes as tested by filtering an ethanol-based dispersion (10 ml) of SiC nanoparticles (0.2 mg/ml) through the membrane (A = 2.23 cm^2^) mounted on a conventional Buchner funnel (P = 60 kPa). (**A**,**B**) Measured flow rate (**A**) and transmitted fraction (**B**) plotted as a function of fractional PTFE/Graphene mass uptake for three types of composite membranes (i.e. those in Fig. 1H–J, see legend in panel (**C**). N.B the transmitted fraction is the fraction of SiC nanoparticles (by mass) transmitted through the membrane. The solid line in A is a fit to Eq. (4). (**C**) Transmitted fraction plotted vs. flow rate for a range of composite membranes with the performance of some commercial membranes shown for comparison. N.B in the plot, high performance membranes should be located toward the bottom right. (**D**) The performance can be approximated by the ratio F/T, which is plotted versus membrane density. The membranes produced here perform extremely well at low density.

Shown in Fig. 3A is flow rate data, measured for GF membranes filled with PTFE-NPs-only as well as PTFE-NPs and graphene at a range of values of ΔM/M_GF_. The latter type of membranes were prepared in two ways; by varying the concentration of graphene in the soak dispersion as well as varying the soak time. In all cases, as ΔM/M_GF_ is increased, the flow rate falls off compared to the GF-only commercial membranes. However, the decrease in flow rate was considerably more rapid in the PTFE-NP/graphene membrane compared to those with PTFE-NPs alone. This suggests graphene to significantly influence the pore structure within the membrane.

We can model this behaviour quantitatively via Darcy’s law which describes fluid flow through porous media. In its simplest form, this gives an equation for the flow rate [m^3^/s], Q^42^:

1 $$Q = \frac{kA\Delta P}{{\eta t}}.$$

where k is the permeability of the porous medium, t and A are its thickness and area, ΔP is the pressure drop across the medium and η is the fluid viscosity. Because of the way we define F ($F = Q/A\Delta P$), we can write $F = k/\eta t$. We would expect the permeability to depend on the typical size of the pores as well as the porosity, P. Modelling the pore distribution as fractal, it has been shown that the permeability is given by^42^

2 $$k = CA_{P} \frac{{P^{m} }}{1 - P},$$

where C is a constant, A_P_ is a measure of the cross-sectional area of the pores and m is the tortuosity factor which lies in the range 1 < m < 4.

In addition, we would expect the porosity to depend on the mass uptake, ΔM/M_GF_. It is straightforward to show that

3 $$P = 1 - \frac{{\rho_{GF - Mem} }}{{\rho_{GF} }} - \frac{{\rho_{GF - Mem} }}{{\rho_{G + P} }}\Delta M/M_{GF} ,$$

where $\rho_{GF - Mem} /\rho_{GF}$ ≈0.026 is the ratio of the density of the glass fibre membrane to that of the glass fibres alone and $\rho_{G + P}$ is the combined density of the graphene/polymer filler.

Combining all of these equations yields

4 $$F = \frac{{CA_{P} }}{\eta t}\frac{{\left[ {1 - \frac{{\rho_{GF - Mem} }}{{\rho_{GF} }} - \frac{{\rho_{GF - Mem} }}{{\rho_{G + P} }}\Delta M/M_{GF} } \right]^{m} }}{{\frac{{\rho_{GF - Mem} }}{{\rho_{GF} }} + \frac{{\rho_{GF - Mem} }}{{\rho_{G + P} }}\Delta M/M_{GF} }}.$$

We have fit this equation to the data in Fig. 3A finding very good agreement once the following fit parameters are used: A_P_ ~ 6 × 10^–14^ m^2^, m = 1, $\rho_{GF - Mem} /\rho_{G + P}$ = 4.1 (assuming C ~ 1/8π)^42^. We can estimate the characteristic pore size from √A_P_ = 250 nm, a value which seems reasonable given the size of the graphene nanosheets. However, we note that this value should be treated with caution as this model cannot account for the inevitable reduction in pore size as ΔM/M_GF_ is increased.

In addition, the value of $\rho_{GF - Mem} /\rho_{G + P}$ is very high, implying the polymer/graphene network to be even sparser than the GF network. However, this discrepancy is almost certainly due to the spatial non-uniformity of the polymer/graphene due to its concentration at the membrane surface. This fit parameter suggests that, averaged over the whole membrane, ρ_G+P_ ~ 50 kg/m^3^. Assuming the true density of the actual polymer/graphene layer is 1000–2000 kg/m^3^, this would imply the polymer/graphene layer to occupy ~ 5–10% of the membrane thickness. From Fig. 1G,H, we can estimate the polymer/graphene layer thickness to be ~ 5–10 μm thick. Because the GF membrane thickness is ~ 500 μm thick, this is ~ 1–2% of the thickness, in reasonable agreement with the estimation above.

Shown in Fig. 3B is a graph of the fraction by mass of SiC particles transmitted through the membrane (T), a parameter which is a measure of the membrane’s selectivity. For the GF membranes infused with PTFE-NPs-only, we find T to be very close to the value of 0.06 measured for the GF-only membranes showing the PTFE nanoparticles to have very little impact on the selectivity. Combined with the flow rate data in Fig. 3A, this implies that although much of the pores are being blocked by PTFE nanoparticles and so reducing the flow rate, some very large pores exist which allow a significant amount of the SiC nanoparticles to pass through the membrane.

However, the GF membranes filled with a combination of PTFE-NPs/graphene show a completely different picture. The transmitted fraction of SiC particles falls dramatically with ΔM/M_GF_, reaching values as low as ~ 10^–4^ for polymer/graphene loadings above 10%. This high degree of selectivity shows that presence of graphene to result in a significant reduction in pore size as ΔM/M_GF_ increases.

Any practical membrane requires high values of F, coupled with low values of T (i.e. high flow rate and high selectivity). To investigate this, we plotted T versus F in Fig. 3C for both GF/PTFE-NP membranes as well as GF/PTFE/Graphene membranes. To put this data in context, we measured F and T for a range of labscale commercial membranes fabricated from teflon, polypropylene, polyester, nylon and cellulose. We find the GF/PTFE/Graphene membranes to have combinations of F and T which are superior to all the commercial membranes tested.

Given that we require high values of F coupled with low T, we can crudely express the performance of the membrane via F/T. This parameter is plotted in Fig. 3D as a function of membrane density. By this metric, Fig. 3D clearly shows the GF/PTFE/Graphene membranes to be far superior to both GF/PTFE membranes and commercial membranes. Interestingly, F/T shows a clear peak for GF/PTFE/Graphene membranes with densities in the range 200–230 kg/m^3^. These densities are just above that of GF-only networks (~ 200 kg/m^3^), implying that the ideal situation involves a very thin surface layer of graphene and PTFE which can block most of the particles without significantly reducing the flow rate.

### Size selectivity

It is likely that it is the smallest SiC nanoparticles which are transported through the membrane with the largest ones becoming trapped. This means that the size of the transmitted particles is likely to be related to the size of the pores within the membrane. In turn, because the PTFE-NP-only membranes appeared to contain large pores, we expect a reduction in pore size as the graphene content is increased. To test this, we filtered a stock SiC/ethanol dispersion through a range of composite membranes prepared from soak dispersions with various values of C_G_/(C_G_ + C_P_) (and so different ΔM/M_GF_). In each case, we measured the transmitted fraction and used TEM to measure the diameter distribution of SiC nanoparticles in the filtrate. The same procedure was carried out in commercial membranes made of PET (nominal pore size 200 nm) and alumina (nominal pore size 50 nm).

**Figure 4:**
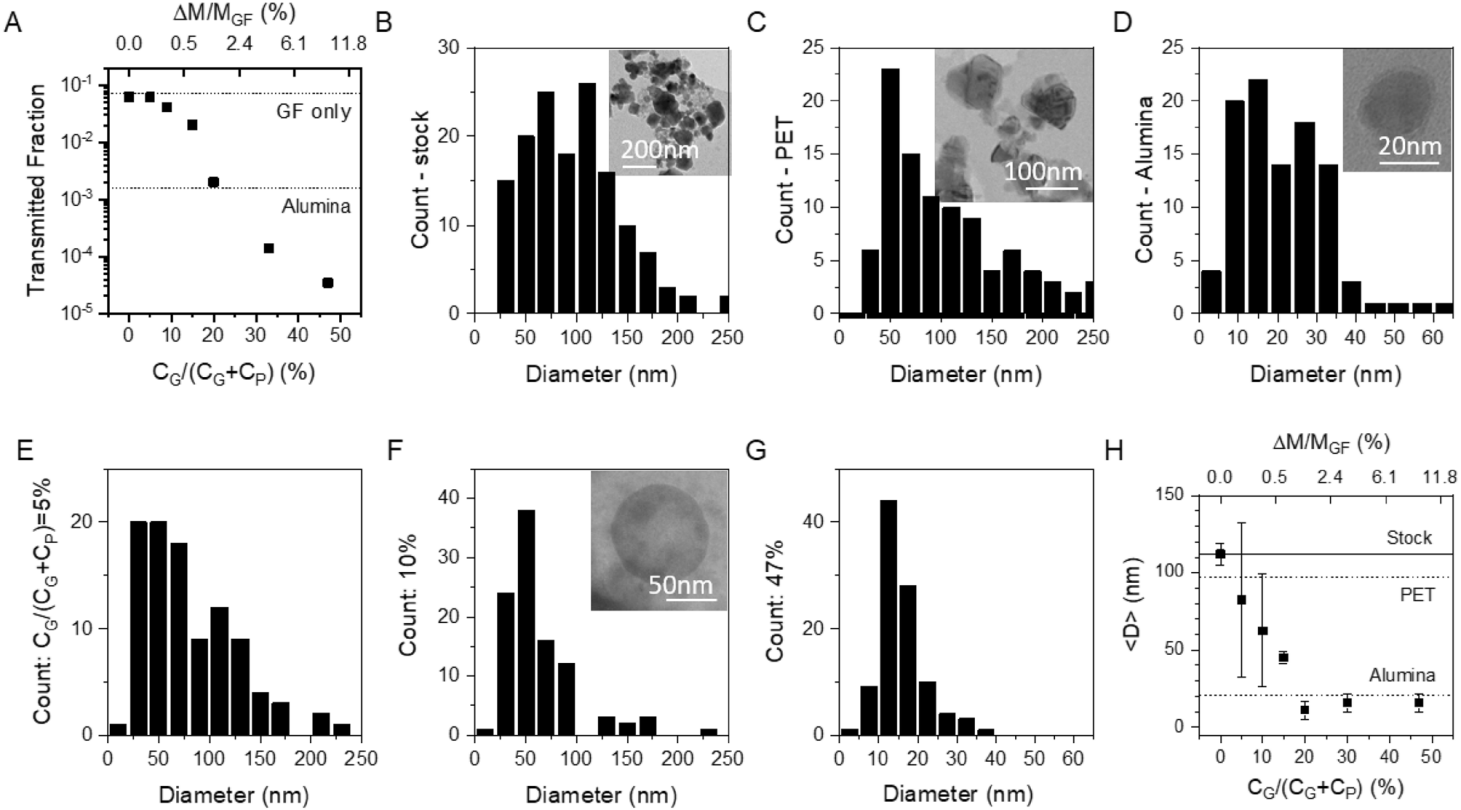
Size of transmitted SiC particles. (**A**) Transmitted fraction as a function of C_G_/(C_G_ + C_P_) as an indicator of nominal graphene content. The top axis is a non-linear scale and shows equivalent values of mass uptake, ΔM/M_GF_. (**B**) Diameter distributions of SiC nanoparticles in the stock dispersion (0.2 mg/ml) used to perform the filtration experiments to probe filter properties. (**C**–**F**) Diameter distributions of SiC particles found in the filtrate (i.e. transmitted through the filter) for stock dispersions filtered through (**C**) a PET membrane (nominal pore size 100 nm), (**D**) an alumina membrane (nominal pore size 20 nm), (**E**–**G**) GF membranes filled with PTFE and graphene from soak dispersions with C_G_/(C_G_ + C_P_) values of (**E**) 5%, (**F**) 9% and (**G**) 47%. The insets in (**B**–**D**,**F**) show sample TEM images. (**H**) Mean transmitted SiC particle diameter plotted versus C_G_/(C_G_ + C_P_) as an indicator of nominal graphene content. The top axis is a non-linear scale and shows equivalent values of mass uptake, ΔM/M_GF_. The solid horizontal line in (**H**) represents the mean SiC particle size in the stock. The dashed horizontal lines in (**H**) represent the mean particle size transmitted through the PET and Alumina membranes. The PET membrane studied represents the polymer membrane with the smallest nominal pore size. In (**A**,**H**) the 0% data point represents a GF membrane infiltrated with PTFE-NPs only.

Shown in Fig. 4A is the transmitted fraction for a subset of composite membranes plotted versus C_G_/(C_G_ + C_P_) of the soak dispersion (estimated ΔM/M_GF_ is given in the top axis). This data is consistent with Fig. 3B and confirms the selectivity to improve dramatically with mass uptake. To test size selectivity, we first used TEM to measure the diameter distribution of the stock dispersion of SiC nanoparticles in ethanol, as shown in Fig. 4B (see insets for typical images). The distribution spans between ~ 25 nm and 250 nm with a mean at 112 nm.

When the stock dispersion is filtered through a membrane, only those nanoparticles small enough to pass through the pores get transmitted to the filtrate. Shown in Fig. 4C is the measured size distribution for SiC nanoparticles filtered through a commercial PET membrane (nominal pore size 200 nm). The distribution is quite similar to the stock (mean of 97 nm) showing that all nanoparticle sizes can travel through such large pores size networks. We performed the same test with alumina membranes (nominal pore size 50 nm). Due to their small pore size, such membranes should only pass very small nanoparticles. However, such high selectivity means these membranes are very expensive (~ €13 per membrane of diameter of 25 mm, www.sigmaaldrich.com). As shown in Fig. 4D, the diameter distribution of the transmitted particles is much narrower than that associated with either the stock or the filtrate from the PET membrane, spanning between ~ 5 and ~ 50 nm with a mean at 21 nm.

The equivalent diameter distributions for nanoparticles transmitted through GF/PTFE/graphene composite membranes is shown in Fig. 4E–G for membranes prepared from soak dispersions with C_G_/(C_G_ + C_P_) values of 5%, 9% and 47% respectively. It is clear from these graphs that the diameter distributions of the transmitted nanoparticles falls sharply as the graphene content in the soak dispersion increases. To show this quantitatively, we measured the SiC nanoparticle diameter distributions for filtrates which passed through a range of membranes prepared from soak dispersions with various C_G_/(C_G_ + C_P_). We have plotted the mean transmitted particle diameter, <D>, versus C_G_/(C_G_ + C_P_) in Fig. 4H. For C_G_/(C_G_ + C_P_) = 0 (equivalent to a GF/PTFE-NP membrane) < D > is virtually identical to that of the stock indicating no particle size selectivity. However, as C_G_/(C_G_ + C_P_) is increases, < D > falls sharply before saturating in the range 7–16 nm for values of C_G_/(C_G_ + C_P_) above 20%. The diameter distribution for the 47% membrane (Fig. 4G) is significantly narrower than that of the alumina membrane and shows no transmission for particles above 40 nm.

This is a significant result: addition of a small amount of graphene and PTFE to extremely cheap GF membranes yields selectivity which is similar to that of much more expensive alumina membranes. Comparing Fig. 4A,H it is clear that alumina-like selectivity performance can be achieved once C_G_/(C_G_ + C_P_) > 20% which is equivalent to ΔM/M_GF_ > 1%. However the significantly higher flow rate under these conditions is a significant advantage. This is an extremely small amount of PTFE/graphene suggesting the conversion of low performance GF membranes to high performance composite membranes to be cost effective.

The fit in Fig. 3A suggests the characteristic pore cross-sectional area to be A_P_ ~ 6 × 10^–14^ m^2^. As discussed above this implies a pore size of √A_P_ = 250 nm, a value that looks too large in light of the data in Fig. 4H which implies pore widths of ~ 20 nm in our systems. We can reconcile this discrepancy by noting that nanosheet networks would be expected to have slit-shaped pores as defined by the gap between two adjacent aligned nanosheets. If we assume such pores have typical length, L_P_, and width, W_P_, we can make the approximation that A_P_ ~ L_P_W_P_. Then taking A_P_ ~ 6 × 10^–14^ m^2^ and assuming W_P_ ~ 40 nm in line with the data in Fig. 4G allows us to estimate L_P_ ~ 1.5 μm, a value which is similar to our mean nanosheet length (~ 0.6 μm). This shows our data to be largely self-consistent.

### Field trials

While the lab-based testing described above suggests the GF/PTFE/graphene composite membranes to be promising ultrafiltration membranes, it is important to demonstrate their efficacy under more realistic conditions. One important application of ultrafiltration membranes is the removal of particulate impurities from dirty water to produce potable water. Clearly, for water purification in poor regions of the world, it will be very important to achieve high selectivity at low cost. Thus, it is important to assess the utility of our composite membranes for water purification.

**Figure 5:**
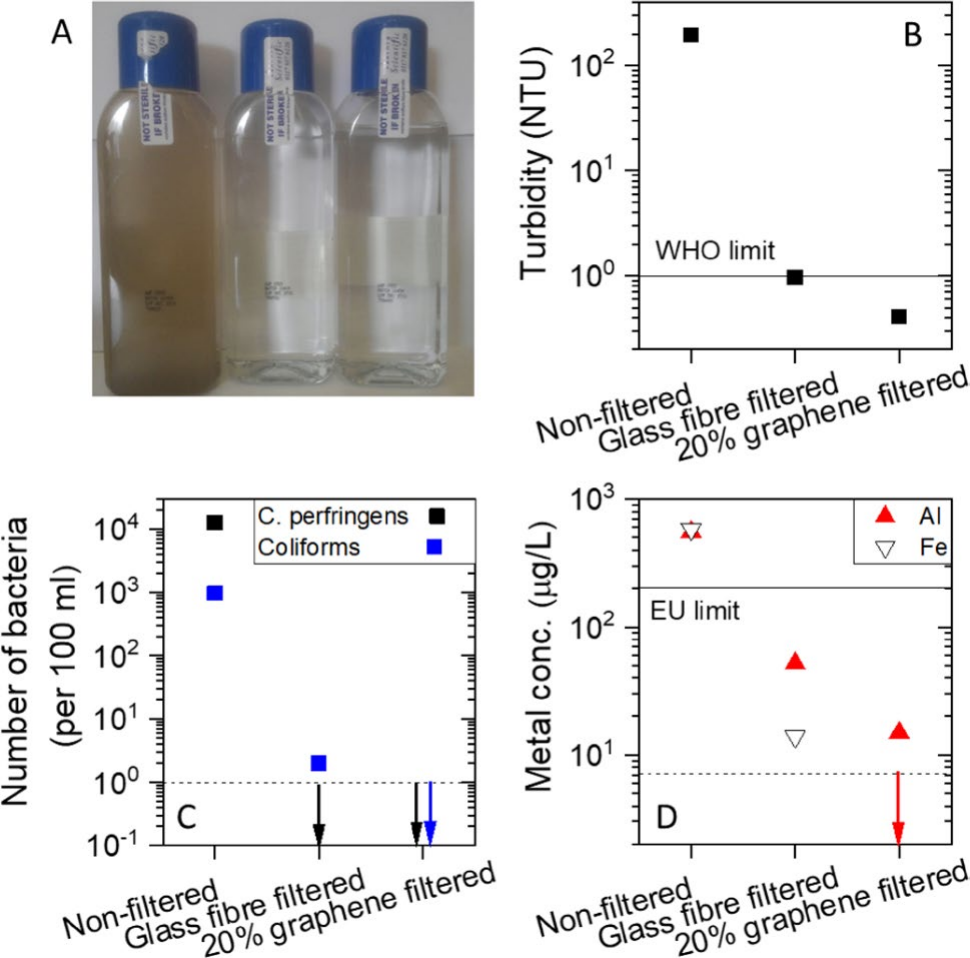
(**A**) Photograph of water collected from the Grand Canal in Dublin before filtration (left), after filtration through a glass fibre membrane (middle) and after filtration through a C_G_/(C_G_ + C_P_) = 20% GF/PTFE/graphene membrane (ΔM/M_GF_ ~ 1.3%, right). (**B**–**D**) Impurity metrics as measured by a commercial water purity testing firm for the three water samples in (**A**). In (**B**), turbidity is a measure of visible light scattering and is commonly used to indicate the presence of impurities in water. The unit used is the Nephelometric Turbidity Unit (NTU). In (**C**), C. perfringens is a bacterium which is a common cause of food poisoning while coliforms are a bacteria group, which are a commonly used indicator of water quality. The number of C. perfringens is reported in colony forming units (CFU) while the number of coliforms is reported as the most probable number (MPN). The solid lines in (**B**,**D**) represent the recommended limits set by the WHO and the EU. In (**D**) the graphene-based membrane data was below the Fe detection limit of the experiment (dashed line) as indicated by the arrow. In (**D**), both the GF and graphene-based membrane data were below the C. perfringens detection limit while the graphene-based membrane datum was below the coliforms detection limit as indicated by the arrows (detection limit shown as dashed line).

To test this, we collected dirty water from the Grand Canal, an inner-city canal in Dublin which is not known for its water purity. The collected water was brown and turbid as shown in the left hand container in Fig. 5A. Portions of the dirty water were filtered through a glass fibre membrane (see Fig. 5A middle) and a C_G_/(C_G_ + C_P_) = 20% GF/PTFE/graphene membrane (ΔM/M_GF_ ~ 1.3%, see Fig. 5A right). After filtration, both samples appeared clean and transparent to the naked eye.

To perform quantitative purity data, we sent both dirty and clean samples to a commercial water testing company (City Analysts Limited) for analysis. Of the parameters tested, probably the most interesting are the turbidity, the bacteria content and the metal (Fe and Al) content.

The turbidity is a measure of light scattered from a liquid and is often used as a measure of suspended solids content. In the as-collected, un-filtered (Fig. 5B) water the turbidity was 196 NTU (Nephelometric Turbidity Unit), ~  × 200 higher than the WHO recommended limit^43^ for drinking water (1 NTU). Filtration through the untreated GF membrane brought the turbidity down to 0.97 NTU, just below the WHO limit. However, filtration through the composite membrane brought the turbidity down even further, to 0.4 NTU. This indicates that the composite membrane is approximately × 2.5 times more effective at removing solids than the GF membrane.

The concentration of two types of bacteria, C. perfringens and coliforms, are given in Fig. 5C. C. perfringens is a bacterium which is a common cause of food poisoning while coliforms are a bacteria group, which are a commonly used indicator of water quality. In the untreated water, the concentrations of C. perfringens and coliforms respectively are 12,600 and 970 units per 100 ml. After filtration through the GF membrane the C. perfringens concentration had fallen below the detection limit (i.e. < 1 per 100 ml) while the coliform concentration had fallen to 2 per 100 ml. However, after filtration through the composite membrane, both bacteria concentrations had fallen below the detection limit. This shows the composite membrane is at least twice as effective at removing coliform as the GF membrane.

Water quality testing also gives the concentration of metal, usually iron and aluminium. In water, iron is found both as Fe^2+^ ions and in the form of compounds such as iron oxides^44^. Similarly, aluminium in the form of a range of compounds, most commonly salts^45^. In the untreated water (Fig. 5D), the concentrations of iron and aluminium were very similar at 583 and 550 μg/L, significantly above the EU recommended limit for drinking water of 200 μg/L^46^. After filtration through the GF membrane the concentrations fell to 14.5 and 52 μg/L respectively. However, after filtration through the composite membrane the iron concentration fell below the detection limit (7.2 μg/L) while the aluminium concentration fell to 15 μg/L. This means the composite membrane is at least twice as effective at removing iron and × 3.5 times as effective at removing aluminium as the GF-only membrane.

## Conclusion

In conclusion, we have demonstrated a method to infuse combinations of liquid-exfoliated graphene nanosheets and water-stable Teflon nanoparticles into commercial glass-fibre membranes. Thermal annealing causes the coalescence of the PTFE, resulting in a mechanically stable structure. These composite membranes are considerably stronger and stiffer than neat glass-fibre membranes and mechanically competitive with commercial membranes. Although the addition of graphene/PTFE does reduce the flow rate compared to the neat glass-fibre membranes, it remains superior to many commercial membranes. However, the selectivity, as measured by the fraction of the probe nanoparticles transmitted through the membrane, increased by × 10^3^ compared to glass-fibres alone. We found the combination of flow rate and selectivity to be far superior to any of the commercial membranes tested under similar conditions. By measuring the diameter distribution of SiC nano-particles remaining in the permeate which was transmitted through the composite membranes, we estimate the mean pore size to be ~ 20 nm, similar to commercial alumina membranes which are relatively expensive. In a field trial we used our membranes to purity dirty canal water. After filtration, we found reductions in turbidity and metal content of almost × 3000 and × 100 respectively to well below international limits and a complete removal of bacteria. We believe this technology represents a potentially low-cost route to reasonably high performance ultra-filtration membranes.

## Methods

Graphite (Sigma Aldrich) was ultra-sonicated using ultrasonic processor UP200S Hielscher at a concentration of 120 mg/ml in TWEEN/water solution (4 mg/ml) for 72 h. The resultant dispersion was centrifuged at 500 RPM for 45 min and the supernatant collected. The graphene nanosheets within the supernatant were collected by filtration through a Sterlitech 0.45 micron PVDF membrane. The resultant filter cake was re-dispersed in deionised water by ultrasonic tip sonication for 1 h at a concentration of 40 mg/ml to yield a stock dispersion. The methods used here are very similar to those previously reported by use for the exfoliation of graphene^47, 48^. In addition, PTFE nanoparticles dispersion was supplied by 3 M Dyneon, (Dispersion TF 5035GZ). The graphene stock dispersion was mixed with the PTFE nanoparticle dispersion in various proportions such that the liquid volume was always 10 ml, the PTFE mass was always 100 mg but the graphene mass varied between 0 and 150 mg. After mixing, these composite dispersions were sonicated for 15 min to homogenise in a low power sonic bath. The graphene/PTFE/water dispersions (10 ml) were then poured into Petri dishes and a pre-weighed glass fibre (www.sterlitech.com) membrane placed in each to soak. After 45 min soaking, the membrane was taken out of the petri dish and placed on a tissue paper for 15 min to remove excess water. The resultant GF/graphene/PTFE membranes were placed in an oven at 50 °C overnight to remove any remaining water and then annealed in a furnace at 420 °C for 45 min to allow the PTFE particles to coalesce. The graphene/PTFE/GF and control membranes were sonicated in ethanol to remove any unbound graphene or PTFE before being dried in ambient conditions for 45 min. Each membrane was then weighted again to yield the mass of graphene + PTFE deposited on the GF. The process yielded a set of graphene/PTFE/GF membranes with various graphene/PTFE ratios.

The membranes were characterised by filtering 10 ml dispersions (0.2 mg/ml) of SiC nanoparticles in ethanol under pressure (60 kPa, controlled via the pump) through the membrane and measuring both the filtration time, to yield the flow rate, and the concentration of SiC in the filtrate, to yield the transmitted fraction. The SiC concentration was measured via the optical absorbance of the dispersion at 550 nm using an absorption coefficient of 13.85 ml·mg^−1^·cm^−1^. The transmitted fraction is defined as the fractional mass of SiC particles which were transported through the membrane. This procedure was repeated three time for each membrane (i.e. each mass fraction) and the flow rate and transmitted fraction data averaged.

The thicknesses of all membranes were measured using digital micrometer with the untreated GF membranes displaying a thickness of 525 ± 25 μm. Diameters were recorded using a digital calipers. Mechanical properties were measured using a Zwick Z0.5 ProLine Tensile Tester (100 N Load Cell) tensile tester with a gauge length of 6 mm and strain rate of 1 mm/min. TEM grids were prepared by first diluting the dispersions and then drop-casting them onto holey carbon grids (Cu 400 mesh). Residual solvent was removed from the grids by drying in a vacuum oven. Bright field TEM images were obtained using a Jeol 2100 operating at 200 keV. Scanning electron microscopy on the composites was undertaken using a Zeiss Ultra Plus. Using the SE2 detector, samples were examined at a working distance of ∼ 2 mm, with a 30 μm aperture and a voltage of 5 kV. Samples for cross-sectional imaging were prepared by freeze fracture. Water purity tests were performed by the commercial testing company City Analysts Limited.
